# Supplementary material for: A S180F substitution in D-alanine aminotransferase confers resistance to β-chloro-D-alanine in Staphylococcus aureus
Source: J Biol Chem. 2025 Nov 11;301(12):110931. doi: 10.1016/j.jbc.2025.110931 (PMC12723384; doi:10.1016/j.jbc.2025.110931)
Supplement: Supporting informatio [file mmc1.pdf]

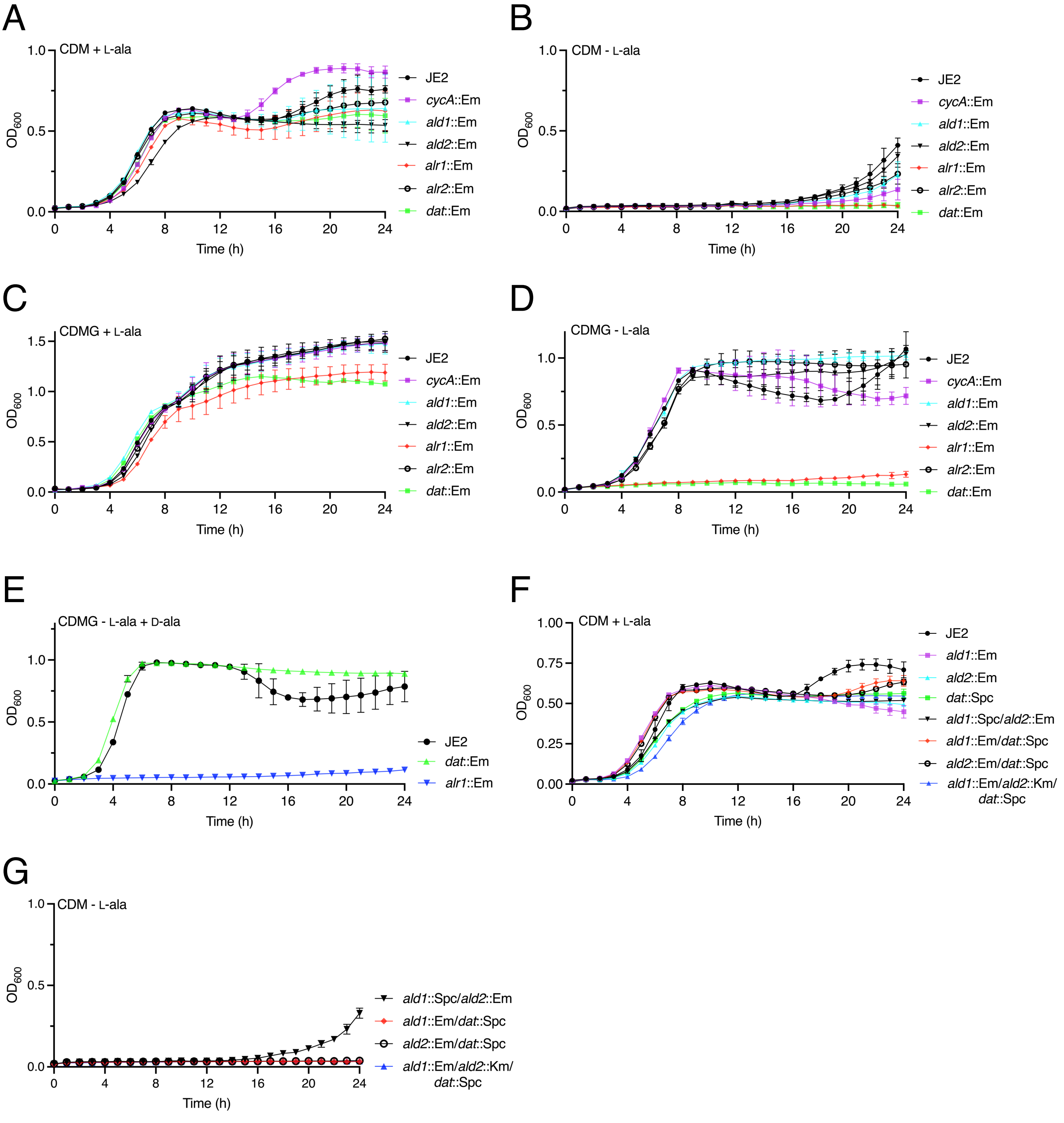

Fig. S1. *S. aureus* is auxotrophic for L-alanine in CDM but not CDMG. A-D. Comparison of JE2, *cycA* (*cycA*::Em^{r}, NE810), *ald1* (*ald1*::Em^{r}, NE1136), *ald2* (*ald2*::Em, NE198), *alr1* (*alr1*::Em, NE1713, *alr2* (*alr2*::Em^{r}, NE799) and *dat* (*dat*::Em^{r}, NE1305) growth in CDM + L-alanine (A), CDM - L-alanine (B), CDMG + L-alanine (C) and CDMG - L-alanine (D). **E.** Comparison of JE2, *alr1* and *dat* growth in CDMG - L-alanine + D-alanine. **F.** Comparison of JE2, *ald1, ald2, dat, ald1*::Spc/*ald2*::Em, *ald1*::Em/*dat::*Spc, *ald2*::Em/*dat*::Spc and *ald1*::Em/*ald2*::Km/*dat::*Spc growth in CDM + L-alanine (G). **G.** Comparison of JE2, *ald1*::Spc/*ald2*::Em, *ald1*::Em/*dat::*Spc, *ald2*::Em/*dat*::Spc and *ald1*::Em/*ald2*::Km/*dat::*Spc growth in CDM - L-alanine. Where indicated L- and D-alanine were added at a final concentration of 5mM. The data presented are the average of at least 3 biological replicates and standard deviations are shown.

A

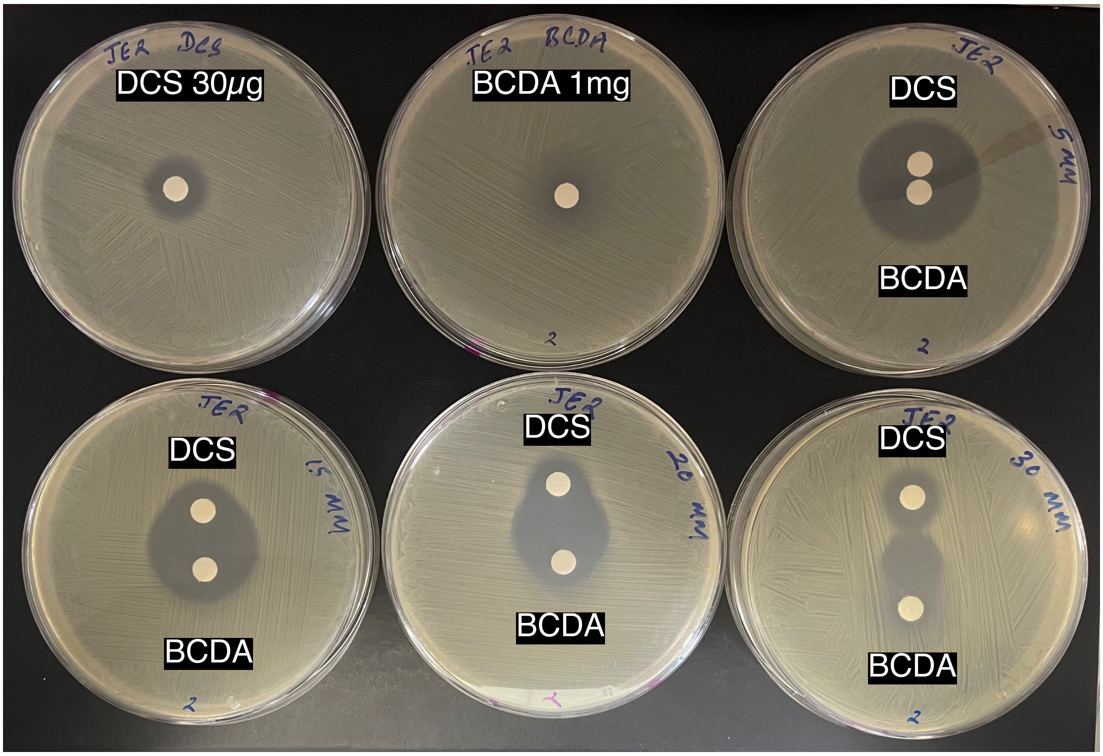

B

DCS (μg/ml)

DCS (μg/ml) \ BCDA (μg/ml)	0	2	4	8	16	32	64	128	256	512	1024	2048
128	0.076	0.075	0.073	0.072	0.074	0.075	0.073	0.074	0.076	0.078	0.077	0.086
64	0.075	0.076	0.075	0.073	0.074	0.074	0.073	0.074	0.076	0.077	0.076	0.08
32	0.073	0.075	0.083	0.074	0.076	0.078	0.074	0.075	0.078	0.078	0.077	0.082
16	0.339	0.233	0.171	0.157	0.083	0.075	0.074	0.074	0.076	0.076	0.077	0.081
8	0.508	0.442	0.407	0.399	0.251	0.075	0.095	0.074	0.077	0.076	0.076	0.079
4	0.502	0.425	0.423	0.457	0.319	0.312	0.088	0.076	0.079	0.079	0.078	0.085
2	0.651	0.455	0.451	0.429	0.422	0.373	0.218	0.087	0.077	0.079	0.079	0.083
0	0.637	0.441	0.428	0.416	0.414	0.438	0.384	0.39	0.298	0.203	0.182	0.083
JE2	0	2	4	8	16	32	64	128	256	512	1024	2048

BCDA (μg/ml)

Fig. S2. DCS and BCDA are synergistic against MRSA. A. Disk diffusion assays with DCS (30 μg) and BCDA (1000 μg) against JE2 grown on Mueller-Hinton agar for 24 h at 37°C. **B.** Checkerboard titration assays conducted using DCS and BCDA with JE2 grown for 24 h at 37°C in Mueller-Hinton broth in 96-well plates. The data shown are the OD_{600} values for each well. The experiments were repeated at least three times and the data from a representative 96-well plate is shown. Red shaded boxes indicated wells in which growth was measured.

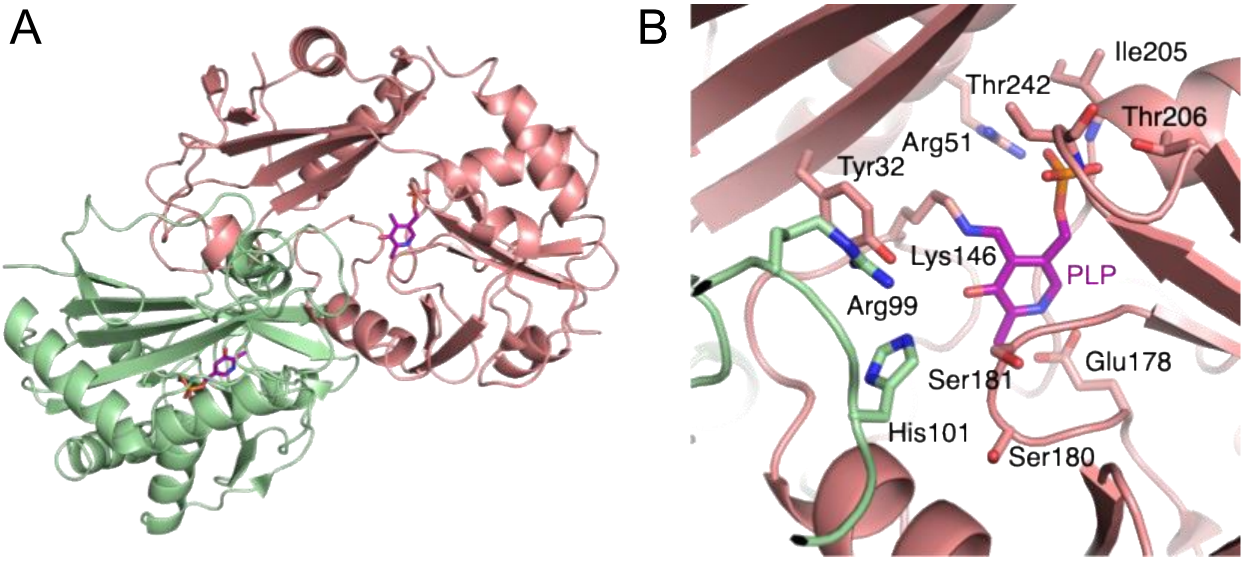

Fig. S3. A. The biological dimer of Dat generated using crystallographic symmetry. **B.** The shared active site of Dat formed by residues contributed from both subunits.

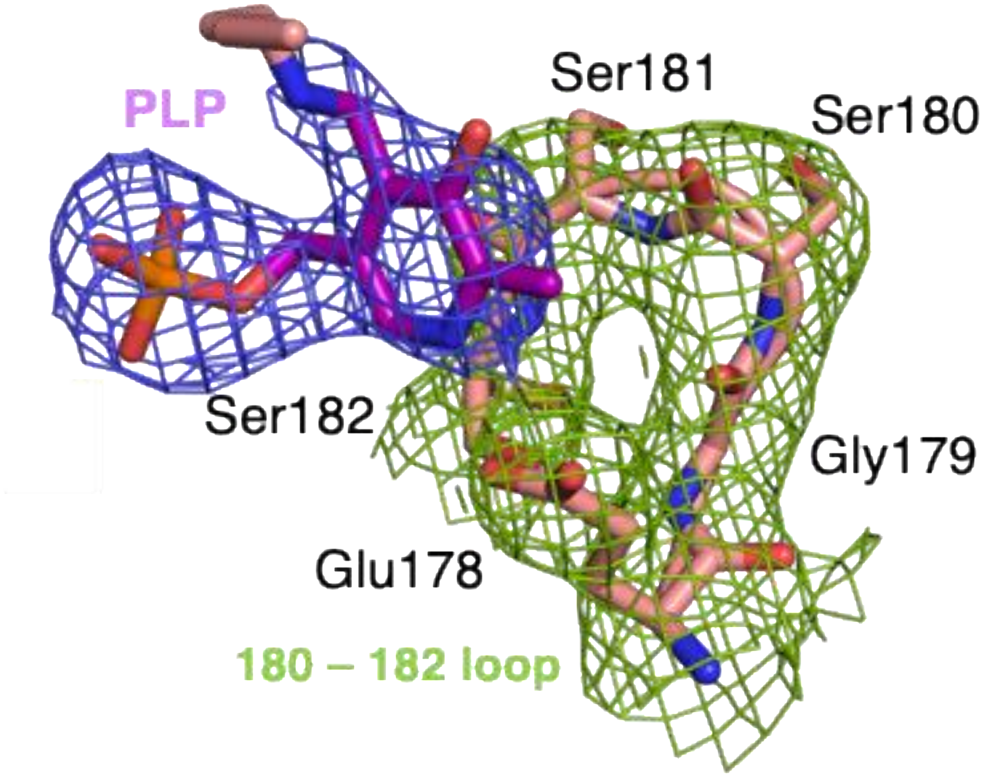

Fig. S4. The calculated 2Fo-Fc omit map indicating PLP and the 180 – 182 loop. The map is contoured at 1σ. The density of PLP is in purple and the map of the loop harboring Ser180 is in green. The carbon atoms of Dat are in tan and the carbons for the PLP are in purple. The oxygen, nitrogen and phosphorous atoms are red, blue, and orange, respectively.

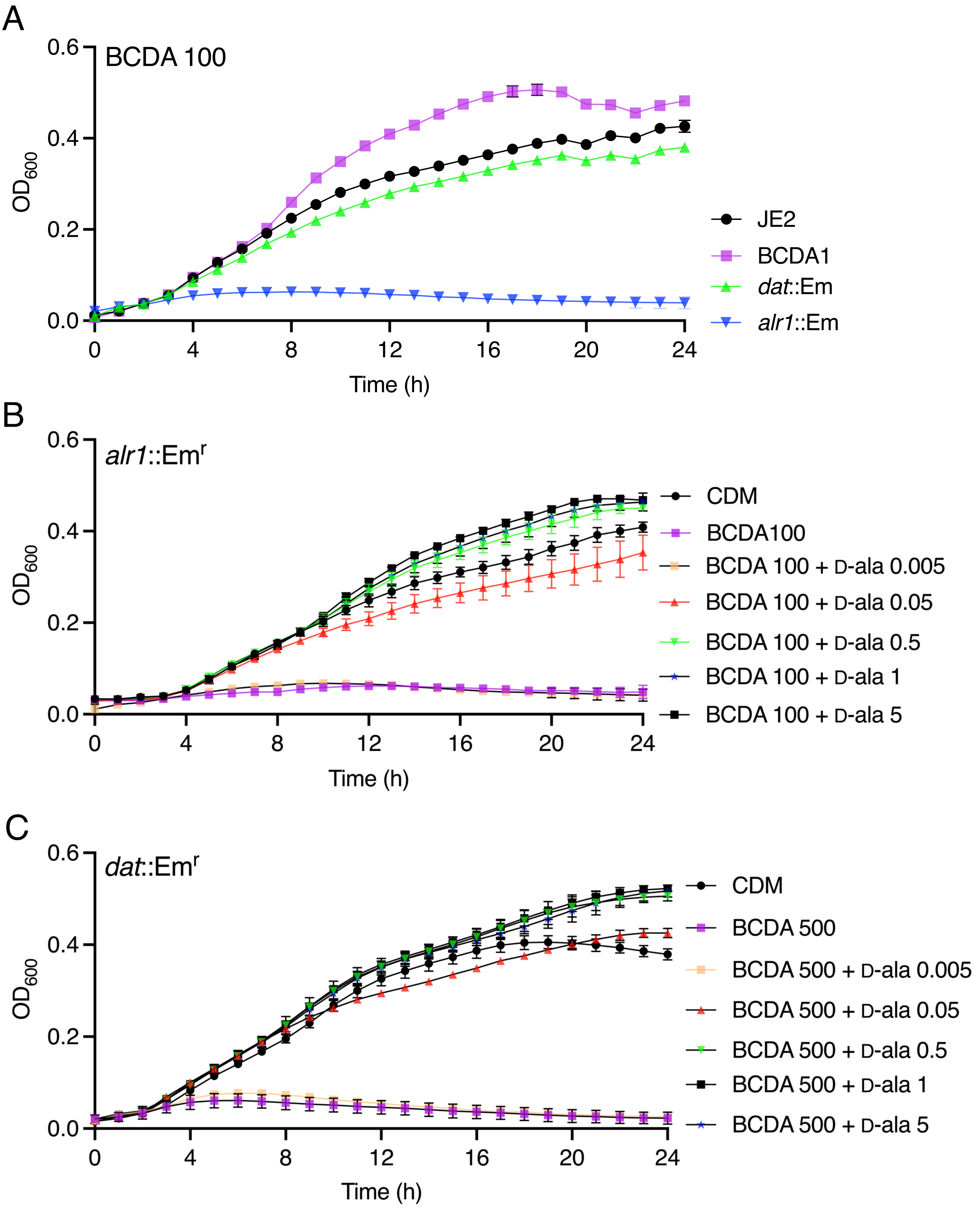

Fig. S5. Exogenous D-alanine restores growth of *alr1* and *dat*::Em^{r} mutants inhibited by BCDA. A. Comparison of JE2, BCDA1, NE1305 (*dat*::Em) and *alr1* growth in CDM supplemented with BCDA 100 μg/ml. **B.** Comparison of *alr1* growth in CDM supplemented with BCDA 100 μg/ml alone or with exogenous D-alanine concentrations from 0.005 to 5 mM. **C.** Comparison of *dat*::Em^{r} growth in CDM supplemented with BCDA 100 μg/ml alone or with exogenous D-alanine concentrations from 0.005 to 5 mM. The data presented are the average of at least 3 biological replicates and standard deviations are shown.

Table S1: Strains and plasmids used in this study

Strains or plasmids	Description	Source
**Strains**		
S. aureus JE2	CA-MRSA, USA300, cured of plasmids. Parent strain of Nebraska Transposon Mutant library (NTML).	(1)
NE810	NTML mutant. *cycA*::Em^r	(1)
NE1713	NTML mutant. *alr1*::Em^r	(1)
NE799	NTML mutant. *alr2*::Em^r	(1)
NE1136	NTML mutant. *ald1*::Em^r.	(1)
NE198	NTML mutant. *ald2*::Em^r.	(1)
NE1305	NTML mutant. *dat*::Em^r	(1)
NE1898	NTML mutant. *pepV*::Em^r	(1)
BCDA1	BCDA resistant derivative of JE2. C-to-T mutation at nucleotide 539 of the *dat* gene (SAUSA300_1696) resulting in a predicted $S_{180}F$ amino acid substitution in Dat.	This study
BCDA1 *dat*::Em^r	*dat*::Em^r transduced into BCDA1 strain	This study
JE2 pLI50	Plasmid pLI50 transformed into JE2	This study
JE2 p*pepV*	Plasmid p*pepV* transformed into JE2	This study
JE2 p*pepV-dat*	Plasmid p*pepV-dat* transformed into JE2	This study
JE2 p*pepV-dat*$_{C539T}$	Plasmid p*pepV-dat*$_{C539T}$ transformed into JE2	This study
BCDA1 p*pepV-dat*	Plasmid p*pepV-dat* transformed into JE2	This study
alr1/dat$_{C539T}$	*alr1*::Em allele transduced into BCDA1 strain	This study
alr2/dat$_{C539T}$	*alr2*::Em allele transduced into BCDA1 strain	This study
dat::Spc	*dat*::Em^r allele in NE1305 swapped for *dat*::Spc	This study
ald2::Km	*ald2*::Em allele in NE198 swapped for *ald2*::Km	This study
alr1::Em/*dat*::Spc	*alr1*::Em allele transduced into *dat*::Spc strain	This study
dat::Spc/*ald1*::Em	*ald1*::Em allele transduced into *dat*::Spc strain	This study
dat::Spc/*ald2*::Em	*ald2*::Em allele transduced into *dat*::Spc strain	This study
dat::Spc/*ald2*::Km	*ald2*::Km allele transduced into *dat*::Spc strain	This study
ald1::Em/*ald2*::Km/*dat*::Spc	*ald1*::Em allele transduced into *dat*::Spc/*ald2*::Km strain	This study
E. coli XL-1 Blue	General plasmid maintenance strain	Agilent Technologies
E. coli BL21 (DE3)	Strain for protein over-expression and purification	Invitrogen
E. coli BL21 (DE3) pET28b	Plasmid pET28b transformed into *E. coli* BL21 (DE3)	This study
E. coli BL21 (DE3) pET28b_Dat	Plasmid pET28b_Dat transformed into *E. coli* BL21 (DE3)	This study

E. coli BL21 (DE3) pET28b_*dat*$_{C539T}$	Plasmid pET28b_*dat*$_{C539T}$ transformed into *E. coli* BL21 (DE3)	This study
E. coli IM08B	*E. coli* DC10B with the staphylococcal (CC8-2)-type methylation system integrated between *atpI* and *gidB* and the (CC8-1)-type methylation system integrated between *essQ* and *cspB.*	(2)
**Plasmids**		
pLI50	*E. coli-S. aureus* shuttle vector	(3)
pET28b	Vector for protein over-expression and purification in *E. coli* BL21 (DE3)	Novagen

References

1. Fey, P. D., Endres, J. L., Yajjala, V. K., Widhelm, T. J., Boissy, R. J., Bose, J. L. *et al.* (2013) A genetic resource for rapid and comprehensive phenotype screening of nonessential *Staphylococcus aureus* genes *mBio* **4**, e00537-00512
2. Monk, I. R., Tree, J. J., Howden, B. P., Stinear, T. P., and Foster, T. J. (2015) Complete Bypass of Restriction Systems for Major *Staphylococcus aureus* Lineages *mBio* **6**,
3. Lee, C. Y., Buranen, S. L., and Ye, Z. H. (1991) Construction of single-copy integration vectors for *Staphylococcus aureus Gene* **103**, 101-105

Table S2: Primers used in this study

Primer name	Sequence (5'-3')	Purpose	Restriction site
**FP_*cycA***	ACAGAATAGCCACAAATAGCACC	*cycA* mutant	-
**RP_*cycA***	GAACTTAATGTCCCAAGCCCT	verification	-
**FP_*ald1***	GAGGTGTTCGACGAATAAATGG	*ald1* mutant	-
**RP_*ald1***	TTCACAAATGAAAGAGGAGTGTTGC	verification	-
**FP_*ald2***	CGCTGTAATCCATTCCCTTT	*ald2* mutant	-
**RP_*ald2***	TGAACCGTCAACTGCGATTA	verification	-
**FP_*dat***	GGATCCGACAAGGGTGTAGCATTTGGC	Verification of BCDA1 strain	*Bam*HI
**RP_*dat***	GAATTCCCTCAACCAATGCCTACATTACACG		*Eco*RI
**FP_Dat**	CGGAATTCGGAAAAAATTTTTTTAAATGGTGAGTTTGTAAGTCC	Recombinant Dat over-expression and purification	*Eco*RI
**RP_Dat**	ACGCGTCGACTTAAATACTGTGTGACTCTATATACTTTTCAAATCCTTC		*Sal*I
**FP_*pepV-dat***	ACGCGTCGACGCCAACTTGCATTGTCTGTAG	Complementation of *dat* mutations	*Sal*I
**RP_*pepV***	CGGAATTCCACTTGGACTTACAAACTCACC		*Eco*RI
**RP_*pepV-dat***	CGGAATTCCCTCAACCAATGCCTACATTACACG		*Eco*RI
**FP_*alr1***	CGGAAAAGCTTCGTTCGCTAGG	*alr1* mutant	**-**
**RP_*alr1***	CAACGACGAACTTGGCAAACC	verification	**-**
**FP_*alr2***	CGCCATTCTTATTTGGGGAAGA	*alr2* mutant	**-**
**RP_*alr2***	TGGTAACGGCGCATCTGTTC	verification	**-**
**FP_T7-promoter**	TAATACGACTCACTATAGGG	pET28b	**-**
**RP_T7-terminator**	GCTAGTTATTGCTCAGCGG	recombinant plasmids	**-**
